# Unique patterns of medial meniscus extrusion during walking and its association with limb kinematics in patients with knee osteoarthritis

**DOI:** 10.1038/s41598-023-39715-0

**Published:** 2023-08-02

**Authors:** Yosuke Ishii, Masakazu Ishikawa, Yuko Nakashima, Takato Hashizume, Saeko Okamoto, Goki Kamei, Kaoru Okada, Kazuya Takagi, Makoto Takahashi, Nobuo Adachi

**Affiliations:** 1 Department of Biomechanics, Graduate School of Biomedical and Health Sciences, Hiroshima University, 1-2-3 Kasumi, Minami-ku, Hiroshima, 734-8553 Japan; 2 Department of Orthopaedic Surgery, Faculty of Medicine, Kagawa University, Kagawa, Japan; 3 Department of Orthopaedic Surgery, Graduate School of Biomedical and Health Sciences, Hiroshima University, Hiroshima, Japan; 4 Ultrasound Business Operations, Healthcare Business Headquarters, KONICA MINOLTA, INC, Tokyo, Japan

**Keywords:** Musculoskeletal system, Rheumatic diseases

## Abstract

Medial meniscus extrusion (MME) is exacerbated by repeated mechanical stress. Various factors would affect MME; however, there is limited information about the behaviour of the medial meniscus during walking in patients with knee osteoarthritis (KOA). This study aimed to investigate the pattern of MME during walking and its association with limb biomechanics in patients with KOA. Fifty-five patients with KOA and ten older adult volunteers as a control group were involved in this study. The MME and limb biomechanics during walking were evaluated simultaneously by ultrasound and a motion analysis system, respectively. The waveform was constructed from the values of MME, and the point showing the highest value of MME was identified during the gait cycle. According to the peak timing of MME in the waveform, the pattern of the waveform was evaluated and compared to the control group. Lateral thrust, knee adduction moment (KAM), and flexion moment were obtained from motion analysis, and their association with the MME was evaluated. The patients with KOA demonstrated unique peak timing during walking. Compared to the control group, there were three groups of MME waveforms, early (< 59%), normal (60–83%), and late (> 84%) from the peak timing in the gait cycle. The pattern of MME waveform in early, normal, and late groups was correlated with the first KAM and lateral thrust, second KAM, and knee flexion moment, respectively. A unique MME pattern during walking was demonstrated, and these patterns were associated with limb biomechanics in patients with KOA.

## Introduction

The menisci function as shock absorbers and distribute the mechanical stress of the knee as a hoop function^1^. However, medial meniscus extrusion (MME) shows meniscal dysfunction and gradually expands^2–4^. The greater MME is associated with the accelerated progression of knee osteoarthritis (KOA) and severe knee pain, leading to poor quality of life^5–7^. Moreover, the greater MME is also a risk factor for a poor clinical score for postoperative patients^8,9^. The minimisation of MME with appropriate management is required to prevent KOA progression at all stages.

The MME temporarily increases in a weight-bearing situation when compared with non-weight-bearing^10,11^. It gradually expands by repetitive mechanical stress during daily activities^2–4^. This increase in MME results in unique behaviour during walking, especially as severe extrusion occurs in the late stance phase on the gait cycle, which is associated with limb mechanical stress^12,13^. However, the lateral thrust and knee adduction moment (KAM) cause repetitive mechanical stress on the medial compartment^14–16^. The association between these mechanical stresses and the behaviour of MME during the gait cycle has not been elucidated yet.

This study aimed to investigate the pattern of meniscal behaviour during walking and its association with limb biomechanics in patients with KOA.

## Material and methods

### Participants

Ethics Committee of epidemiology of Hiroshima University approved this study (Approval Number: E449-4). This study is in accordance with the principles of the Declaration of Helsinki. All participants gave their informed consent.

From April 2021 to May 2023, 70 patients with KOA and ten healthy older volunteers (control group) participated in this cross-sectional study (mean age, KOA: 62.5 ± 10.6 years, males, n: 35; control: 61.4 ± 8.0 years, males, n: 6), and they were all able to walk smoothly. The patient with KOA in the medial compartment was diagnosed radiographically. The severity of KOA was evaluated with Kellgren–Lawrence (K/L) score using the Rosenberg view. Their knee alignment was evaluated by the hip-knee-ankle angle (HKAA) on the images of the full lower limb. The inclusion criteria were (1): HKAA < 0°, (2): Knee pain and tenderness in the medial joint space, and (3): Awareness of knee pain during activities of daily living, especially loading tasks.

If participants had the following, (1): Pain and tenderness in the lateral compartment, (2): A history of trauma, (3): A neurological disorder, (4): A need for any support due to severe knee pain, which affected the gait form, they were excluded. The final KOA group comprised 55 knees in 55 patients (Table 1).

**Table 1:** Demographic data of participants.

	Control subjects	KOA patients
Knees	10	55
Gender (M:F)	6 : 4	24 : 31
Age (years)	61.4 ± 8.0	61.6 ± 9.4
BMI (kg/m^2^)	22.6 ± 2.2	25.4 ± 3.4*

Values represent means ± standard deviation.

*BMI* body mass index.

*Means the significant higher within group (p < 0.05).

### Gait analyses

The kinematic data were obtained from a three-dimensional motion analysis system (VICON612; Vicon Motion Systems, Oxford, UK) with sixteen cameras (Vicon Motion Systems) at 100 Hz. Moreover, eight force platforms (AMTI, Watertown, Mass) at 1000 Hz were synchronised with the motion analysis system and obtained the kinematical data. In the process, the cameras were calibrated, and passive reflex makers were attached to the participant's lower leg according to the model of the Plug-in-Gait Marker (Plug-in-Gait, Vicon® Peak; Vicon Motion Systems). The participants walked 5 m on a straight course at a comfortable speed. The gait cycle was identified as the heel contact and toe-off using the threshold of vertical ground reaction force 10N and determining the stance phase in the walking cycle as being from heel contact to toe-off. The stance phase's kinetics, kinematic and spatiotemporal data were analysed by the Vicon Nexus (Vicon® Peak; Vicon Motion Systems). The Lateral thrust was calculated as the difference in knee angle between the varus and valgus occurring in the early stance phase of the gait cycle^17^.

### Dynamics of the meniscus and its behaviour of MME during walking

**Figure 1:**
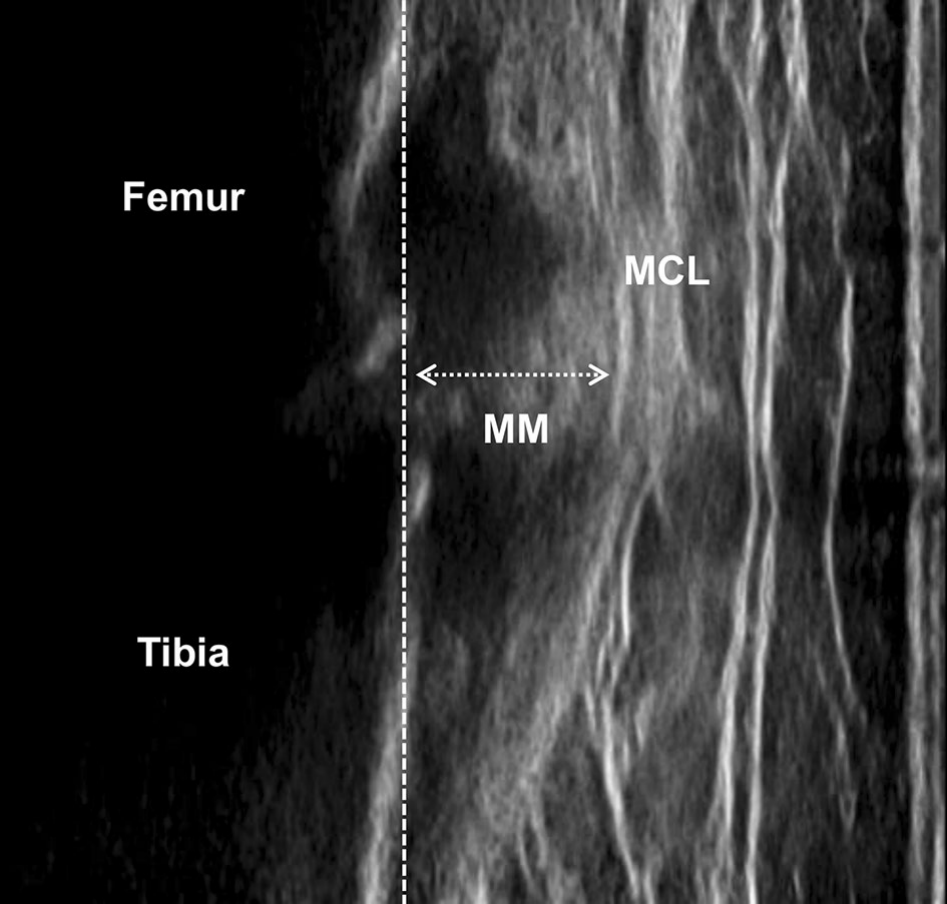
The evaluation of meniscus extrusion on ultrasound. *MM* medial meniscus, *MCL* medial collateral ligament. The dashed line shows the cortex of the medial tibial plateau. The extruded medial meniscus is shown as the dashed arrow line.

The dynamic meniscus extrusion was obtained by ultrasonography (SNiBLE, KONICA MINOLTA, Japan) and a prototype 3–11 MHz specific liner transducer. The procedure is described in previous studies^12,18^. Briefly, the longitudinal transducer was placed on the medial joint space where the medial collateral ligament and the triangle meniscus can be seen on the ultrasound images (Fig. 1). Secondly, the transducer was fixed using a flexible band which adapts to knee flexion during walking and allows for natural walking. The participants were asked to walk the 5 m at a comfortable speed. The meniscus movement during walking was recorded using the video mode on the ultrasound with 30 Hz. The external devices recorded the stimulation and synchronised it with the motion analysis system.

**Figure 2:**
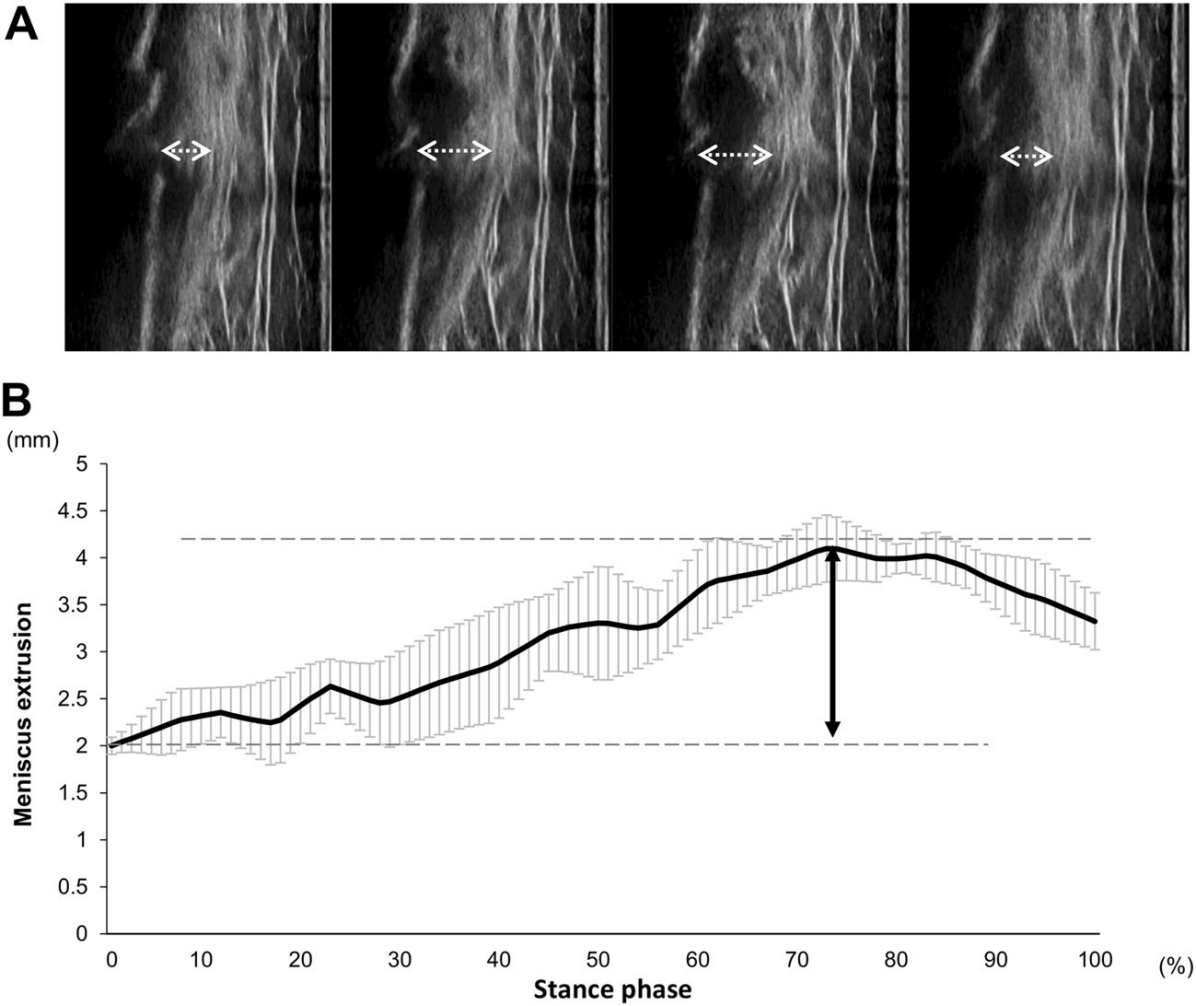
The ultrasound images and the waveform of meniscus extrusion in the gait cycle. These are four representative images of 20, including the extruded meniscus during walking (**A**). The constructed waveform from the serial value of meniscus extrusion on 20 images. The stance phase was standardised and time-normalized to the 101 points data (**B**). The arrow line shows the amount of change in meniscus extrusion (∆MME).

The medial meniscus was identified as “The distance from the cortex of the medial tibial plateau to the remotest point of the medial meniscus” based on a previous study^19^, and the value of MME was calculated using Kinovea software (v0.8.15; Kinovea open source project, www.kinovea.org) (Fig. 1). The ΔMME was calculated as the difference in MME between the maximum and minimum. These processes have been demonstrated to be highly reliable in a previous study^12^. They continued until around 20 images were obtained, the value of MME in the stance phase of the gait cycle was calculated, and a waveform of MME was made. To compare the different raw data sets on a single stance phase, all data were standardised and time-normalised to the 101 points data (Fig. 2). The behaviour timing was also calculated as the maximum MME in a single stance. Lastly, these processes were repeated three times and the averages were used for statistical analysis.

### Categorisation of the pattern of meniscal behaviour

**Figure 3:**
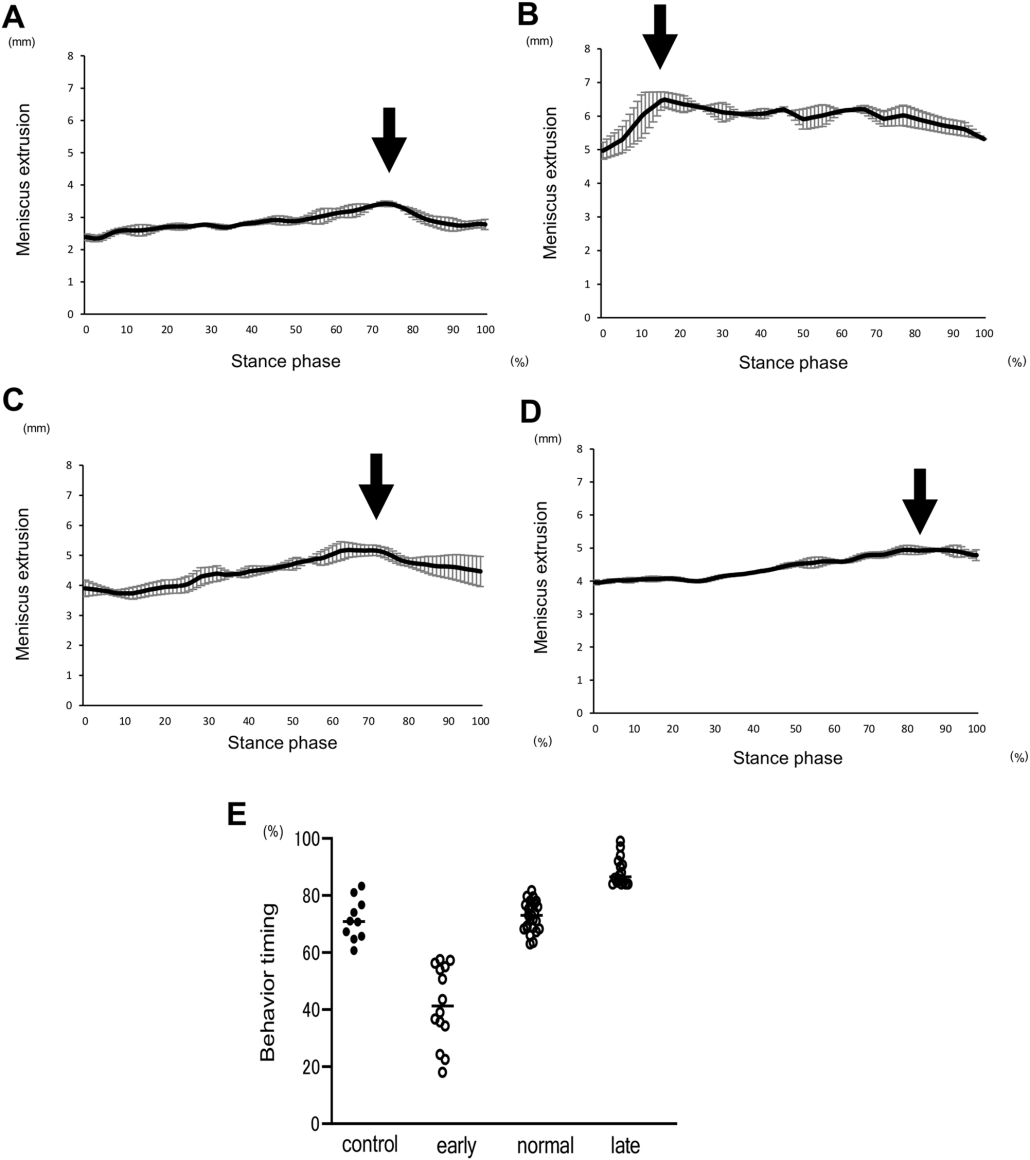
Three patterns of waveforms based on the dynamics of MME and its peak timing. Individual waveforms show the representative patterns of the dynamic meniscus as control (**A**), early (**B**), normal (**C**), and late (**D**) groups. The peak timings in each group are indicated by an arrow based on the maximum extruded meniscus in the stance phase (**E**). The distribution of peak timing in each patient from control, early, normal and late groups.

According to the range of the behaviour timing in the control group, the KOA patients were divided into three groups, (1) the normal group, which was similar to the control (2) the early group that showed extrusion earlier than the controls, and (3) the late group that demonstrated a later peak time (Fig. 3).

### Evaluation of the type of meniscal tear

The meniscus quality was evaluated by magnetic resonance images (MRI), obtained within three months of the first consultation. Two orthopaedic surgeons (M.I. and G.K), each with over ten years of experience, performed the assessments. The type of meniscus tear was identified by the globular or linear signal abnormality within the meniscus that extended to the surface on more than two consecutive images based on previous studies^20–23^ and is then divided into horizontal, longitudinal, radial, complex, and degenerative tears. The meniscus tear on the posterior horn of the root was categorised as a medial meniscus posterior root tear (MMPRT) and exhibited the specific radiological signs; the ghost sign or giraffe neck sign^24,25^.

### Statistical analysis

To compare the groups, the demographic data, MME, and ΔMME were analysed using the Wilcoxon signed-rank test. The demographic data, MME, ΔMME, and biomechanical data within the groups were compared using a one-way analysis of variance or the Kruskal–Wallis test. The correlation between ΔMME and biomechanical data was performed by the Pearson or Spearman correlation method. The presence of MMPRT analysed using Chi-squared test and the multiple comparison was demonstrated in each group. These statistical analyses were performed using SPSS (v23, IBM, USA). A P-value of < 0.05 was considered significant.

## Results

### Demographic data and meniscus quality

The BMI in KOA was higher than that in the control group (p < 0.01). Other parameters were not significantly different between groups (Table 1).

### Comparison with the MME and behaviour of timing, and categorised to group

In KOA, the minimum and maximum MME and ΔMME were significantly greater than those in the control group. (ΔMME, KOA: 1.5 ± 0.6 mm, control: 1.0 ± 0.1 mm; p < 0.01). The behaviour timing in KOA patients varied compared to that in the control group (KOA: 69.4 ± 19.2% range: 18–99%, control: 71.3 ± 6.6%, range: 60–83%).

The KOA patients were comprised of 14 patients in the early (25%), 25 in the normal (45%), and 16 in the late group (29%) according to the behaviour timing in the control group (Behavior timing: early: < 59%, normal: 60–83% and late: > 84%) (Fig. 3E). The average behaviour timing in maximum MME was 41.8 ± 13.3%, 72.6 ± 5.1%, and 88.4 ± 4.7% in the early, normal, and late groups, respectively.

The KOA patients had varus alignment, and almost all patients had mild to moderate disease (HKAA: -5.2 ± 3.3°) (KL I: 6, II: 28, III: 19, IV: 2). In the group, the diseased knee and knee alignment are shown in Table 2. The HKAA in the early group was lower than that in the late group. The maximum MME in the early group was higher than in the late group. However, other parameters did not differ significantly among the groups. These data are summarised in Table 2.

**Table 2:** The demographic data of three groups based on peak timing.

	Early	Normal	Late
Knees (n)	14	25	16
Gender (M:F)	7 : 7	11 : 14	6 : 10
Age (years)	62.7 ± 7.0	60.4 ± 11.2	62.6 ± 7.7
BMI (kg/m^2^)	26.0 ± 2.7	25.0 ± 3.6	25.7 ± 3.6
Minimum MME (mm)	5.0 ± 1.7	3.8 ± 1.7	3.8 ± 1.4
Maximum MME (mm)	6.5 ± 2.0*	5.4 ± 2.1	5.0 ± 1.3
ΔMME (mm)	1.6 ± 0.6	1.6 ± 0.6	1.3 ± 0.3
HKAA (°)	− 7.0 ± 3.2*	− 5.5 ± 2.8	− 3.2 ± 3.1
K/L (I, II, III, IV)	1, 3, 9, 1	2, 15, 7, 1	4, 9, 3, 0

The normal group shows features similar to the control concerning behaviour timing. The earlier or later groups show earlier and later occurring times compared to the control group. The values represent the mean ± standard deviation. *Indicates the significant difference between the early and late groups (p < 0.05).

*MME* medial meniscus extrusion, *ΔMME* the difference in medial meniscus extrusion between maximum and minimum.

### The comparison with gait parameters and biomechanical data

The gait speeds were 0.8 ± 0.1 m/s, 0.9 ± 0.2 m/s and 0.9 ± 0.1 m/s in the early, normal and late groups, respectively. The gait parameters did not differ among the groups, including gait speed, the first peak angle of flexion and varus, and moments during gait (Table 3).

**Table 3:** The gait parameters in each subgroup.

	Early	Normal	Late	p-value
Lateral thrust (°)	1.1 ± 0.6	1.3 ± 0.9	1.4 ± 0.9	0.729
Maximum varus angle (°)	2.5 ± 5.8	3.4 ± 3.7	1.8 ± 6.1	0.634
Maximum flexion angle (°)	21.6 ± 11.3	16.0 ± 5.7	15.4 ± 6.4	0.24
First adduction moment (Nm/kg)	0.5 ± 0.2	0.5 ± 0.1	0.5 ± 0.2	0.469
Second adduction moment (Nm/kg)	0.5 ± 0.2	0.4 ± 0.1	0.4 ± 0.2	0.27
Flexion moment (Nm/kg)	0.6 ± 0.2	0.7 ± 0.2	0.6 ± 0.2	0.49
Impulse of adduction moment (Nms/kg)	0.2 ± 0.1	0.2 ± 0.1	0.2 ± 0.1	0.079
Impulse of flexion moment (Nms/kg)	0.3 ± 0.1	0.2 ± 0.1	0.2 ± 0.1	0.52
Gait speed (m/s)	0.8 ± 0.1	0.9 ± 0.2	0.9 ± 0.1	0.737

The values represent the mean ± standard deviation. *p*-value shows the difference among groups using a one-way analysis of variance or the Kruskal–Wallis test.

### The correlation between meniscal behaviour and biomechanical factors

In the KOA patients, the second KAM tended to correlate positively with ΔMME, but there was no significant correlation (r = 0.21, p = 0.124).

In the early group, the ΔMME was significantly correlated with lateral thrust, first KAM and its impulse. Moreover, the ΔMME was significantly correlated with the second KAM in the normal group, and there were significant correlations with flexion moment and its impulse in the late group (Table 4).

**Table 4:** The correlations between ΔMME and biomechanical factors in each group.

	Early group	Normal group	Late group
Lateral thrust	0.71*	0.29	− 0.48
First knee adduction moment	0.58*	0.25	− 0.05
Second knee adduction moment	0.53	0.44*	− 0.15
Knee flexion moment	0.27	0.04	0.64*
Impulse of knee adduction moment	0.62*	− 0.32	− 0.19
Impulse of knee flexion moment	0.31	− 0.002	0.57*

ΔMME, is the difference in medial meniscus extrusion between maximum and minimum. Lateral thrust; the difference between varus and valgus occurring in the early stance phase in the gait cycle; values represent a correlation coefficient with ΔMME and * indicates a significant correlation (p < 0.05).

### The evaluation of the meniscus quality

The most frequent meniscus pathology included the degenerative tear (42%) in patients with KOA, and a medial meniscus posterior root tear (MMPRT) was found in 17 patients (31%). In the groups, the rate of MMPRT was 12%, 36%, and 56% in the normal, early, and late groups. The presence with and without MMPRT was significantly difference in only normal and late group (p < 0.05). However, there was not difference among groups for other rate of meniscus type (Table 5).

**Table 5:** Type of meniscal tear in KOA patients and distribution in groups.

Type of tear	Early group, n = 14	Normal group, n = 25	Late group, n = 16
Normal intensity	1 (7)	1 (4)	0 (0)
Longitudinal tear	0 (0)	1 (4)	0 (0)
Horizontal tear	2 (14)	4 (16)	1 (6)
Radial tear	1 (7)	1 (4)	1 (6)
Complex tear	0 (0)	1 (4)	1 (6)
Degenerative tear	5 (36)	14 (56)	4 (25)
MMPRT	5 (36)	3 (12)	9 (56)

Values represent n and incidence rate (%).

*MMPRT* medial meniscus posterior root tear.

## Discussion

Our findings show that patients with KOA have various meniscal behaviour patterns individually associated with limb biomechanics.

Meniscus extrusion is a risk factor for the progression of KOA, knee pain, and poor postoperative clinical outcome^6–9^. The increase in extrusion is believed to be due to the greater ΔMME being affected by abnormal mechanical stress. However, each individual with KOA has complex pathology, and the appropriate approach must be based on the knee pathology^26^. The behaviour of the medial meniscus is shown as an effect of mechanical stress and the key factor associated with the mechano-pathology of KOA progression^12,18^. Our findings indicated that the pattern of meniscal behaviour varied during walking, and the groups differed regarding the incidence of MMPRT. Moreover, the biomechanical factors associated with the ΔMME changed according to the severity of the posterior portion of the medial meniscus pathology. Thus, it is important to evaluate the mechano-pathology of KOA, emphasising the pattern of meniscal behaviour. Therefore, our findings provide insight into the mechanisms of ΔMME by mechanical stress in individuals with KOA with meniscal pathology of posterior segment and lead to developing the appropriate approach for KOA patients.

In patients with KOA, a variation in behaviour timing of MME was confirmed compared with the control group. Furthermore, the change in extrusion (ΔMME) showed no significant correlation with mechanical stresses. On the other hand, in three groups consisting of subjects with different behaviour timing, the first KAM and lateral thrust in the early group, the second KAM in the normal group, and flexion moment in the late group were significantly correlated with the ΔMME. Thus, these results might show that KOA results in variable behaviour from MME with specific biomechanical stresses. A previous study showed that only the second KAM was correlated with the ΔMME; however, this data came from a small sample and presumably did not investigate the variety of MME patterns in subjects^12^. To date, the lateral thrust and first KAM are known as the major mechanical stresses on the medial compartment and are known to cause the progression of KOA^14,16,27,28^. The knee flexion moment is a mechanical stress on the anterior compartment of the knee^29^. However, a previous study has reported the effect of the combination of KAM and KFM on the prediction of medial contact force and showed a high predictive value when compared with only KAM^30^. Moreover, the occurring the injury pattern of traumatic MMPRT was investigated and occupied the motion with low knee flexion under loading conditions such as walking and descending actions as the major event^31^. These facts might support that the late group showed the association between ∆MME and KFM and the highest incidence of MMPRT. Thus, these previous studies indicate that KOA patients have complex mechanical pathology, which may explain the plausible relevance of mechanical loading according to the meniscus dynamics in this study.

The meniscal hoop function requires the fixed attachment of the posterior root segment^32^. However, these often show pathological changes such as stretch and tear on the posterior segment of the medial meniscus, leading to loss of the hoop function^33,34^. Our findings demonstrate the incidence of MMPRT was higher in the late and early groups than in the normal group. Moreover, these groups were divided based on the dynamics of the meniscus. Thus, the difference in meniscus dynamics waveform might be reflected in the progressive pathological condition of the posterior section of the meniscus. Typically, the meniscal movement during knee flexion translates not only to the posterior but also within the joint on the tibial plate, whereas the MMPRT shows destruction of the general translation^35–38^. Walking includes the two flexion points in a single gait cycle's early and late phases in the stance phase. The waveform in the normal group was in line with this movement, which occurs in the middle-to-late phase, excluding the early and late phases. On the other hand, early and late groups were unmatched with normal meniscal movement. Therefore, these results and previous studies might explain the correlation between the dynamics of the meniscus and the absence of hoop function associated with meniscal attachment.

This study has some limitations. First, the study was cross-sectional, so our finding cannot determine whether the biomechanical factor occurs because of the ΔMME. Secondly, the sample size was small, so we cannot compare with bias the K/L stage and knee alignments in the groups. Third, the MMPRT was evaluated only with the MRI images. However, MMPRT has several types with partial or complete tears. Therefore, future studies are needed to add the MMPRT arthroscopy information and assess the correlation between matched K/L and knee alignment in a cohort study.

## Conclusion

Unique patterns of MME during walking and their associations with mechanical stress were demonstrated in KOA patients. The evaluation of the features of meniscus dynamics could contribute to the understanding of the mechano-pathology of KOA (Supplementary Information S1, S2).

## Supplementary Information

Supplementary Information 1.

Supplementary Information 2.

Supplementary Legends.

## Data Availability

The datasets generated during and/or analysed during the current study are available from the corresponding author upon reasonable request.
