# Supplementary figures and images for: Unique patterns of medial meniscus extrusion during walking and its association with limb kinematics in patients with knee osteoarthritis
Source: Sci Rep. 2023 Aug 2;13:12513. doi: 10.1038/s41598-023-39715-0 (PMC10397274; doi:10.1038/s41598-023-39715-0)

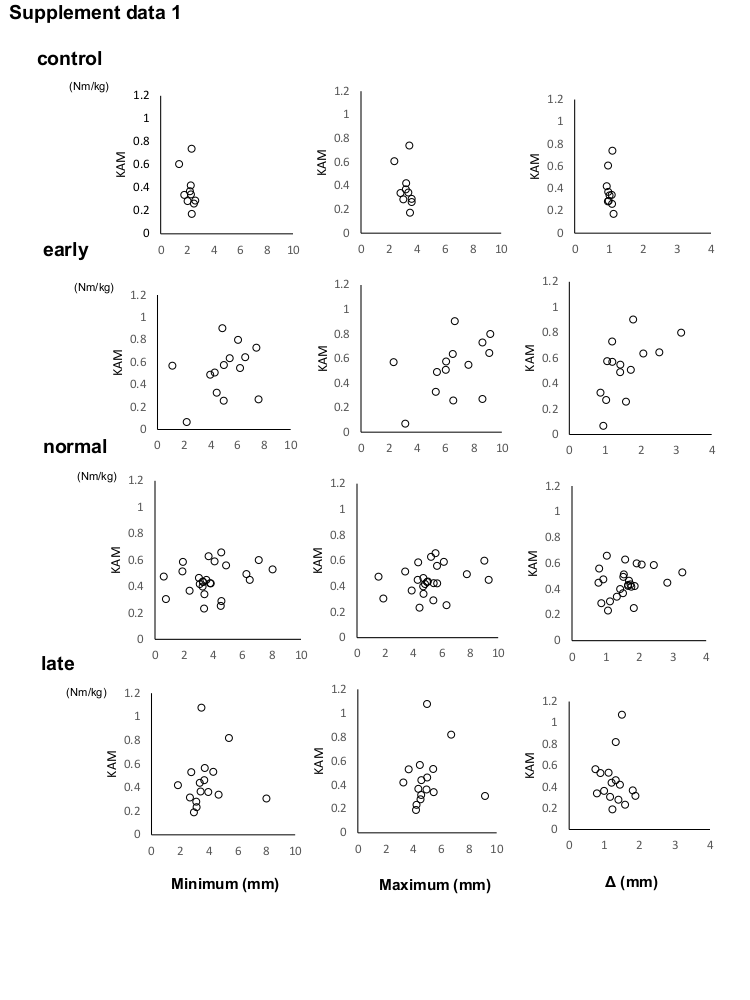

Supplement: Supplementary file 1 — Supplementary Information 1. [file 41598_2023_39715_MOESM1_ESM.tif]

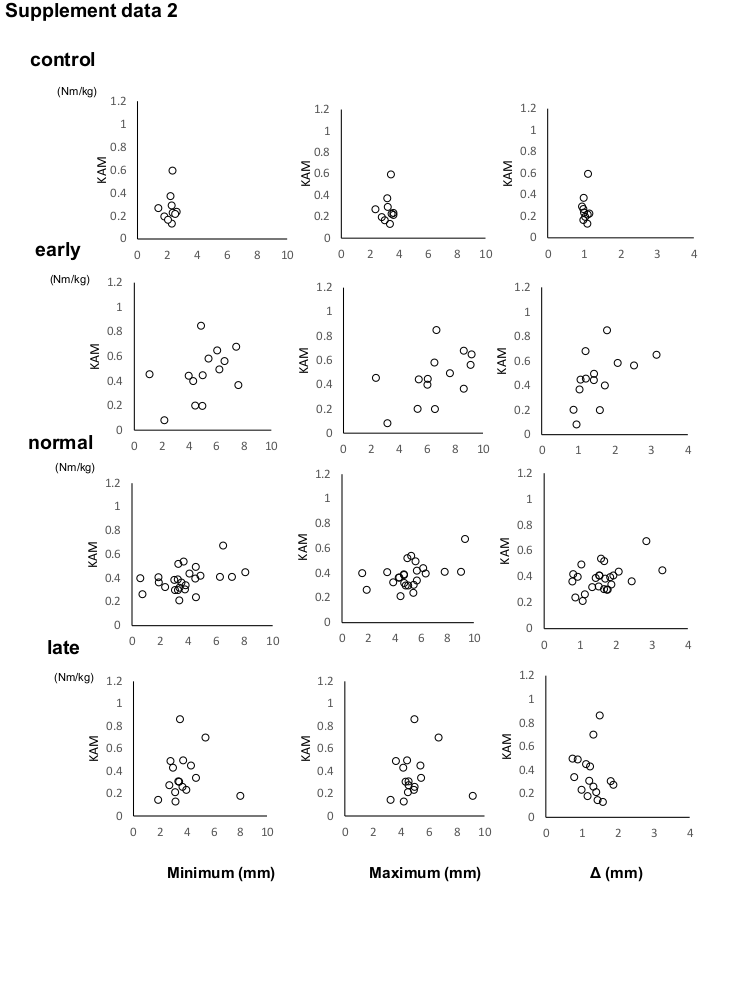

Supplement: Supplementary file 2 — Supplementary Information 2. [file 41598_2023_39715_MOESM2_ESM.tif]
