# Supplementary material for: Unique patterns of medial meniscus extrusion during walking and its association with limb kinematics in patients with knee osteoarthritis
Source: Sci Rep. 2023 Aug 2;13:12513. doi: 10.1038/s41598-023-39715-0 (PMC10397274; doi:10.1038/s41598-023-39715-0)
Supplement: Supplementary file 3 — Supplementary Legends. [file 41598_2023_39715_MOESM3_ESM.docx]

**Supplements legend**

**Supplement data 1**.

The correlation between first KAM and MMEs

These plots show the correlations with first KAM. KAM: knee adduction moment, MME: medial meniscus extrusion, Δ: the difference in medial meniscus extrusion between maximum and minimum.

**Supplement data 2**.

The correlation between second KAM and MMEs

These plots show the correlations with second KAM. KAM: knee adduction moment, MME: medial meniscus extrusion, Δ: the difference in medial meniscus extrusion between maximum and minimum.
